# Growth and Identification of Bacteria in *N*-Halamine Dental Unit Waterline Tubing Using an Ultrapure Water Source

**DOI:** 10.1155/2011/767314

**Published:** 2011-12-15

**Authors:** Nuala Porteous, Jie Luo, Monica Hererra, John Schoolfield, Yuyu Sun

**Affiliations:** ^1^Department of Comprehensive Dentistry, The University of Texas Health Science Center at San Antonio, San Antonio, TX 78229, USA; ^2^Biomedical Engineering Program, University of South Dakota, Sioux Falls, SD 57107, USA; ^3^Department of Microbiology and Immunology, The University of Texas Health Science Center at San Antonio, San Antonio, TX 78229, USA

## Abstract

This study examined bacterial growth and type on biofilm-controlling dental unit waterline (DUWL) tubing (T) and control manufacturer's tubing (C) in a laboratory DUWL model using ultrapure source water that was cycled through the lines. Sections of tubing lines were detached and examined for biofilm growth using SEM imaging at six sampling periods. Bacteria from inside surfaces of T and C, source unit, and reservoir were cultured and enumerated. At six months, organisms were molecularly identified from the alignment matches obtained from the top three BLAST searches for the 16S region. There was a 1–3 log increase in organism growth in a clean, nonsterile reservoir within an hour. Biofilm was established on the inside surfaces of C within three weeks, but not on T. *Proteobacteria*, and *Sphingomonas* spp. were identified in the source reservoir and C line, and a variation of the genera was found in T line.

## 1. Introduction

The presence of bacterial biofilms on the inside of dental unit waterlines (DUWLs) has been well documented and recognized as an undisputed source of contamination of dental patient treatment water [1]. Furthermore, as most DUWL treatment methods have limitations, biofilms are challenging to eliminate [2]. Numerous studies have shown that DUWL biofilms harbor a diverse population of organisms and at least forty genera of bacteria have been identified at the molecular level [3–5]. Although earlier identification techniques were culture-based, certain organisms, such as *Pseudomonas* spp. and *Sphingomonas *spp., have been commonly identified in studies across the globe [5–9]. The phylogenic group *α*-*Proteobacteria* has been shown to be the predominant survivor in chlorinated water distribution systems and *Sphingomonas *spp. are closely aligned with these genera [10].

The majority of studies on DUWL biofilm tested dental units that used source water from the municipal water supply [11–13]. Some studies tested units with source distilled water and demonstrated that distilled water alone did not prevent biofilm formation without a concurrent, regular intermittent DUWL cleaning scheme [14, 15].

No previous studies have reported on biofilm growth when Type I ultrapure water is used as source water. This type of water has dissolved solids in parts per billion (ppb) and is recommended for use for washing/rinsing semiconductor components during manufacture and sensitive laboratory analytical procedures [16].

The purpose of this study was to examine organism growth and type, and biofilm development on the inside surfaces of a biofilm-controlling *N*-halamine DUWL tubing compared with generic manufacturer's polyurethane tubing using ultrapure source water. The biofilm-controlling properties of the *N*-halamine tubing using source tap water have been confirmed in previous work by the authors and have been published elsewhere [17, 18].

## 2. Materials and Methods

Testing was performed using a modified laboratory DUWL model delivery system, first described by the American Dental Association (ADA)/American National Standards Institute (ANSI) working group [19]. Details on the model setup in our laboratory have been described previously [20]. Five days a week for a six-month period, 1,500 mL water was collected from a nanofiltration/uv water purifier (Barnstead NANOpure Diamond Water Purification System) in a clean, nonsterile collection flask, transferred to a nonsterile polycarbonate reservoir, and pumped through two 5 ft-long sections of silicon tubing, and then to *N*-halamine test (T) and generic manufacturer's control (C) lines. T and C lines were comprised of 24 × 5 cm sections that were connected together with 2 cm sections of silicon tubing. Effluent emitted from the lines drained into covered glass collection flasks. A computerized system (Cole Parmer Masterflex System) was used to set the daily flow rate from the reservoir at 1.4 mL/min, 5 min on and 25 min off to simulate a typical workday at a predoctoral teaching dental clinic. 

### 2.1. Laboratory Sampling

There were six sample collection periods; 1 through 4 were done at three-week intervals (Weeks 3, 6, 9, 12); 5 and 6 at 6-week intervals (Weeks 18 and 24). At the beginning of each collection period, water in the reservoir was refreshed and run through T and C lines for five minutes to ensure its distribution. This 5-minute cycle was repeated and laboratory procedures were performed according to standard procedures [21] as follows. 

#### 2.1.1. Sampling from Source Unit, Reservoir, and Inside Tubing Surfaces

- One hundred milliliters (100 mL) of water from source water purifier was collected in a sterile collection container containing sodium thiosulfate (Idexx Lab. Ltd., UK) and refrigerated.
- Three sections of tubing were detached from T and C lines (6 × 5 cm sections). Adherent bacteria inside the six sections were dislodged and suspended in phosphate buffer solution (PBS) by pushing a sterile needle through the lumen and rinsing with PBS into a sterile tube.
- A section of tubing from T and C lines was detached (2 × 5 cm sections), each placed in a fixative of formaldehyde and transported to the Electron Microscopy Laboratory at the UTHSCSA, where both sections were cut and prepared with hexamethyldisilazane (HMDS) for scanning electron microscopy (SEM) imaging of inside surfaces to detect the presence of biofilm [22].
- After Steps (A), (B), and (C) were completed, a 100 mL-sample of reservoir water was collected in a sterile container as described in (A).
- Ten-fold serial dilutions of (A), (B), and (D) samples were made with PBS and one-tenth of a milliliter (0.1 mL) volume of each solution was cultured in triplicate on R2A agar plates, using the spread-plate method. Organisms were incubated at 20–28°C for 7 days, averaged, and reported as mean CFU/mL.
- The daily 8 hr pump cycle was restarted.

### 2.2. Statistical Analysis for CFU/mL Data

Three tubing section samples were analyzed for T and C at the end of each 3- or 6-week pure water exposure period. For comparisons of T and C tubing samples, two-sample Student's *t*-tests were performed to determine if the means of log CFU/mL were significantly different for T and C at the end of each 3- or 6-week period. In addition, the treatment tubing means observed for the 6 time periods were compared to determine if overall study treatment differences were significant. For all comparisons, *P* < 0.05 was considered significant.

### 2.3. Molecular Identification

At Week 25, after bacterial colonies were enumerated, isolates on R2A agar plates were submitted to the Department of Microbiology and Immunology at the UTHSCSA for molecular identification. To determine the etiologic agent, a sequence-based approach using the 16s ribosomal DNA regions as targets for the molecular identification isolates was performed [23]. 

#### 2.3.1. DNA Isolation

Isolates were grown for 12 h at 37°C in R2A agar. A loopful was taken from each plate and suspended in 600 *μ*L cell lysis buffer (Promega, blood Maxwell LEV kit) in a 0.5 mL microfuge tube. The suspension was bead-beaten for 45 seconds to 1 minute to aid in cell wall breakdown and then incubated with proteinase K at 56°C for 15 min. The suspension was then pelleted for 3 min at maximum speed in a microfuge according to the manufacturer's instructions. The supernatant was transferred to the Maxwell LEV cartridge and then mounted on the automated Maxwell system, resulting in 150 ng/*μ*L of purified bacterial DNA after a 45 min run.

#### 2.3.2. Polymerase Chain Reaction (PCR)

PCR reactions were performed directly on 3 *μ*L of the DNA supernatant in a 50 *μ*L reaction using 5 prime PCR Extender system (Fisher Scientific Company, LLC) according to the manufacturer's instructions. 16S amplicons were obtained using primers (27F, 1525R) [23]. PCR conditions were performed as previously described in 5 Prime Extender Fisher manual. Amplifications were performed in a PTC-100 thermocycler (MJ Research, Watertown, Mass, USA) using the preprogrammed, three-step protocol as the standard program for all reactions and consisted of thirty-five cycles using an annealing temperature of 55°C and 1 minute extension time. A 5 *μ*L aliquot of the PCR reaction was run on a 0.7% agarose gel and stained with ethidium bromide to confirm amplification. The remaining PCR reaction (45 *μ*L) was run on a gel, as described above and was gel purified using the Wizard SV Gel and PCR Clean-Up System (Promega, Madison, WI, USA) and eluted in 30 *μ*L sterile H_2_O according to the manufacturer's instructions.

#### 2.3.3. Sequencing

DNA obtained from the PCR reaction was prepared for sequencing by cleaning with Qiaprep Spin Miniprep Kit (Qiagen, Valencia, Calif, USA), according to manufacturer's instructions. The purified DNA was sequenced at the UTHSCSA Advanced Nucleic Acids Core facility. Sequences were then used to perform individual nucleotide-nucleotide searches of the ribosomal 16S region using the BLASTn algorithm at the NCBI website (http://www.ncbi.nlm.nih.gov/BLAST/). Identifications were calculated based on a percentage made from the alignment matches obtained from the top three BLAST searches for the 16S region to yield a variety level identification. The three highest percent identities for each isolate were analyzed for bacterial identification.

## 3. Results

### 3.1. Bacterial Counts

As seen in Table 1, source water bacterial counts measured directly from purifier unit were zero, but increased exponentially when contained in a reservoir for 45–60 minutes during laboratory sampling procedures (A)–(C).

**Figure 1:**
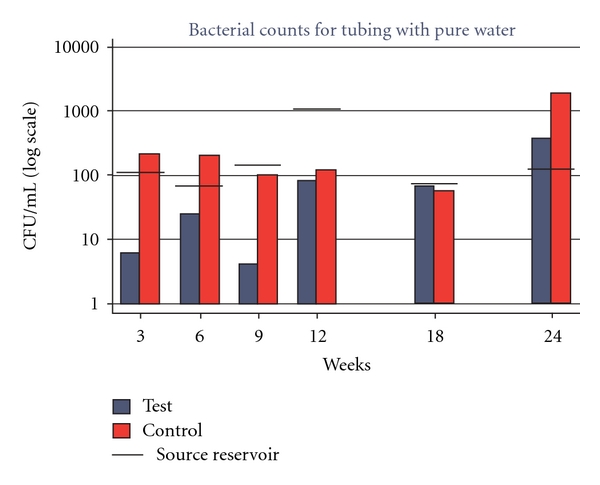
Geometric mean CFU/mL bacteria found in T and C lines and source reservoir.

A three-log increase in bacterial counts was noted in C tubing by the end of six months, and bacterial counts inside T tubing were consistently below the CDC recommended level of 500 CFU/mL (Table 1, Figure 1).

Overall for 3 to 24 weeks, mean log CFU/mL was not significantly different for C compared to T (*t* = 2.09, *P* = 0.063), as seen in Table 2. For individual weeks, C was significantly greater than T at Weeks 3 (*t* = 3.48, *P* = 0.025), 9 (*t* = 2.78, *P* = 0.050), and 24 (*t* = 3.69, *P* = 0.021), but no significant C versus T log mean differences were observed for the other individual week comparisons. 

### 3.2. SEM Imaging

**Figure 2:**
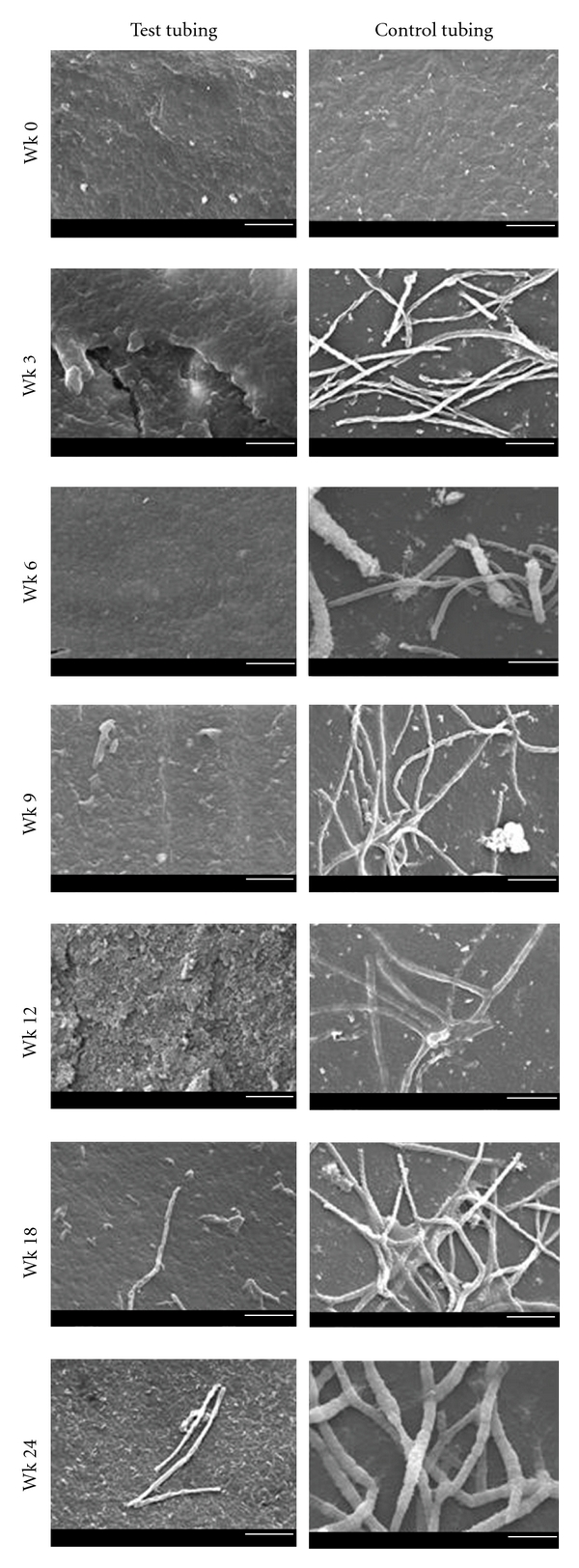
SEM (magnification ×5,000) images showing biofilm growth and development over the 24-week study period.

Within three weeks, SEM imaging showed bacterial proliferation and biofilm establishment on the inside surfaces of C tubing with no microscopically visible bacteria on the inside surfaces of T tubing, as seen in Figure 2. Some scattered bacteria were visible on the inside surfaces of T tubing at the end of the study period.

### 3.3. Organism Identification

BLASTn results for the 16S ribosomal bacterial region returned the following highest % identities.

**Test tubing**

1. isolate 25B1 *Sphingomonas *spp. Identities 977/977 (100%);
2. isolate 25B2 *Blastobacter *spp. 956/956 (100%);
3. isolate 25B2b *Erythromonas ursincola* 956/956 (100%);
4. isolate 25B2c *Sphingomonas natatoria* 956/956 (100%);
5. isolate 25B-3 *Erythromonas ursincola* 1369/1389 (99%);
6. isolate 25B-3b *Sphingomonas natatoria* 1369/1389 (99%).

**Control tubing**

(7) isolate 27A-1 *Sphingomonas *spp. 1350/1352 (99%),
(8) isolate 27A-1b *Proteobacterium symbiont* 1350/1352 (99%),
(9) isolate 27A-2 *Sphingomonas *spp. 982/982 (100%);
(10) isolate 27A-3 *Sphingomonas natatoria* 977/980 (99%).

**Source reservoir**

(11) isolate 8C-1 *Sphingomonas *spp. 980/980 (100%),
(12) isolate 8C-2 *Proteobacterium symbiont of Nilaparvata lugens *959/960 (99%).

## 4. Discussion

This study showed that water organisms grew exponentially within an hour when Type I ultrapure water was contained in a clean, nonsterile, polycarbonate reservoir bottle that was refreshed at the beginning of every working day. Organism growth originated in the clean, nonsterile collection flask, or the reservoir, or both, with subsequent biofilm formation on the inside surfaces of untreated control DUWL tubing. Early biofilm colonizers were well established on the control tubing by Week 3, as seen on SEM images.

Bacterial levels cultured from *N*-halamine tubing remained within the EPA Drinking Water Standard/CDC recommended level of 500 CFU/mL throughout the study period. Previous research by the authors showed that bacterial levels observed for *N*-halamine tubing at each time interval were significantly correlated with the corresponding bacterial levels in source water, with a three-week carry-over effect in T after the source water levels returned to acceptable levels. The authors attributed this to multiplication of organisms in stagnant water inside T, even without biofilm formation [18]. The results of this study seem to confirm those earlier findings as an increase in bacterial levels in Weeks 12, 18, and 24 occurred after an increase in source water CFUs in Week 12 (Figure 1). These findings again highlight the need for ensuring delivery of high quality source water through dental unit waterlines as water samples and cultures are merely a snapshot of bacterial activity at one point in time since monitoring of water quality is not done in between sampling periods.

SEM imaging showed that there was no biofilm formation throughout the study period and no apparent bacterial growth on *N*-halamine tubing until Week 24, although isolated organisms were visible at Week 18. One of the factors known to affect biocidal efficacy is contact time with the bacteria [24]. The biocidal properties of* N*-halamine, which are rechargeable, are due to a chlorine exchange with the contact microorganisms [25]. In this study, the active agent, covalently-bound chlorine, may have been consumed during the course of the six months, thus exhausting its antimicrobial properties and indicating the need for a chlorine recharge before 24 weeks. One of the limitations of the current study is the 6-month duration, and further studies will evaluate the effects of recharging at Week 24 to regenerate the biocidal effects.

It is also possible that those bacteria captured on SEM images at Week 24 may have already expired and been expelled as planktonic bacteria. Identification on cultured, live isolates only was performed in this study, whereas it is necessary to collect and process dead microorganisms, or organisms with low CFU counts directly from the water source. This study limitation also inhibited our ability to confirm, with molecular sequencing, other organisms visible on SEM images. With more novel methods of DNA extraction and better diagnostic selective species-specific probes to detect multiple organisms by Real Time PCR analysis, it will be possible to accurately detect and quantify microorganism growing in these biofilms more accurately without having to rely on culture identification alone in future studies.

Other researchers have demonstrated that a gene mutation or overexpression can result in biocidal resistance when a biocidal agent is used at low concentrations [26]. A previous study identified bacterial isolates that were resistant to sodium hypochlorite and the majority of organisms belonged to the *Proteobacteria *genera [27]. In this study, *Proteobacteria* spp. and *Sphingomonas* spp. that were identified in the source reservoir sample were also isolated from inside the control tubing, whereas a greater diversity of bacterial species belonging to the phylogenic *Proteobacteria* group were isolated from inside the *N*-halamine tubing. Another limitation of this study was the failure to test chlorine resistance of each isolate at different time points, as described previously by Martin et al. [28].

## 5. Conclusions

Type I ultrapure water from a nanofiltration- /uv-treated water purifier that was collected in a nonsterile flask became contaminated after transfer to a reservoir within an hour, and within a six-month period, formed a dense biofilm on the untreated control waterline. The biofilm-controlling *N*-halamine test tubing prevented biofilm formation throughout the study period. However, some scattered organisms were visible on the test tubing by the end of the study period and were identified as a variation of the genera *Proteobacteria* found in the source carboy. This may be explained by one or all of the following reasons.

1. the biofilm-controlling properties of the *N*-halamine test tubing may have become exhausted by the end of the study period and should have been recharged within that time period;
2. the organisms may have become resistant to chlorine and undergone an ecological adaptation in the *N*-halamine tubing during the study period.

Further research over a longer period of time, using ultrapure source water contained in a treated antimicrobial reservoir before delivery to *N*-halamine test tubing is necessary. Identification and chlorine resistance of organisms growing on *N-*halamine tubing over a longer period of time is also necessary using novel methods of DNA extraction. This process may provide clues to ecological adaptation of organisms and ultimately pave the way for a solution to the problem of dental unit waterline contamination.

## Figures and Tables

**Table 1:** Geometric mean number of bacteria (CFU/mL) in Source Reservoir and inside surfaces of test and control lines.

Week	Source purified water unit	Source reservoir water	Test tubing	Control tubing
3	0	1.08 × 10^2^	5.10 × 10^0^	2.08 × 10^2^
6	0	6.84 × 10^1^	2.39 × 10^1^	2.03 × 10^2^
9	0	1.39 × 10^2^	3.20 × 10^0^	1.01 × 10^2^
12	0	1.08 × 10^3^	7.84 × 10^1^	1.19 × 10^2^
18	0	7.27 × 10^1^	6.78 × 10^1^	5.68 × 10^1^
24	0	1.22 × 10^2^	3.79 × 10^2^	1.91 × 10^3^

**Table 2:** Logarithmic mean CFU/mL of bacteria dislodged from inside surfaces of test and control tubing.

Week	Treatment	*N*	Log mean	Log Std Dev	Geometric mean	*t*-value	*P* value
3	Test	3	0.784	0.719	5.1	3.48	0.025
	Control	3	2.321	0.263	208.3	Test < control	

6	Test	3	1.397	0.338	23.9	1.73	0.159
	Control	3	2.310	0.851	203.4		

9	Test	3	0.627	0.737	3.2	2.78	0.05
	Control	3	2.007	0.443	100.7	Test < control	

12	Test	3	1.900	0.747	78.4	0.37	0.729
	Control	3	2.079	0.374	119.0		

18	Test	3	1.838	0.923	67.8	0.09	0.936
	Control	3	1.762	1.229	56.8		

24	Test	3	2.580	0.296	379.4	3.69	0.021
	Control	3	3.280	0.144	1905.6	Test < control	

All Weeks	Test	6	1.521	0.738	32.2	2.09	0.063
	Control	6	2.293	0.526	195.5		
